# Mechanical transfer of *Theileria orientalis*: possible roles of biting arthropods, colostrum and husbandry practices in disease transmission

**DOI:** 10.1186/s13071-016-1323-x

**Published:** 2016-01-22

**Authors:** Jade Frederick Hammer, Cheryl Jenkins, Daniel Bogema, David Emery

**Affiliations:** Faculty of Veterinary Science, University of Sydney, Sydney, NSW Australia; Elizabeth Macarthur Institute, New South Wales Department of Primary Industries, Menangle, NSW Australia; The Ithree Institute, University of Technology Sydney, Broadway, NSW Australia

**Keywords:** *Theileria orientalis*, Ikeda, Theileriosis, Transmission, Mechanical, Iatrogenic, Sucking louse *Linognathus vituli*, Colostrum

## Abstract

**Background:**

The intracellular protozoal parasite *Theileria orientalis* has rapidly spread across South-eastern Australia, substantially impacting local cattle industries since 2006. *Haemaphysalis longicornis* appears to be a biological vector in the endemic regions. Mechanical transfer of blood by biting arthropods, in colostrum or iatrogenic transmission though husbandry procedures is another possible mode of transmission. This study assesses the risk of these mechanical modes of transmission.

**Methods:**

Blood was collected from a *T. orientalis* Ikeda positive Angus steer, and was inoculated into the jugular vein of 9 calves in 3 treatment groups, each with 3 animals. Calves in Group 1 received 10 ml of cryopreserved blood, while those in Groups 2 and 3 received 1 ml (fresh blood) and 0.1 ml (cryopreserved), respectively. An additional three animals remained as negative controls and the donor calf was also followed as a positive control. Blood was collected over 3 months, and analysed via qPCR for the presence of the parasite. Samples of the sucking louse *Linognathus vituli* were collected opportunistically from calves 5 months after inoculation and tested for *T. orientalis.* For the colostral transmission study, 30 samples of blood and colostrum were collected from cows at calving in an endemic herd. These samples along with blood from their calves were tested by qPCR for *T. orientalis* and for antibodies to the major piroplasm surface protein (MPSP).

**Results:**

Eight of the nine inoculated calves became positive for *T. orientalis.* The prepatent period of these infections was inversely correlated with inoculation dose. All negative control calves remained negative and the positive control calf remained positive.

Samples of *L. vituli* tested positive for *T. orientalis* Ikeda, while some samples of colostrum were also shown to be qPCR and anti-MPSP positive. All calves in the colostral study tested qPCR negative although one was antibody-positive.

**Conclusions:**

*T. orientalis* is capable of being mechanically transferred by intravenous inoculation with small volumes of blood and is detectable up to 5 months post-infection. Animals infected by this means may play a significant role in the transmission of the disease by acting as asymptomatic carriers. Other modes of blood transfer, including biting arthropods and colostral transfer are also possible modes of disease transmission.

## Background

Outbreaks of disease associated with the blood-borne intracellular parasite *Theileria* have been seen in Australia since 2006. Although *Theileria* has been recognised in all mainland states in Australia since 1910 [1, 2], it had long been considered a benign parasite [1, 3–5]. However, pathogenic genotypes are now recognised in Australia with a large number of outbreaks of clinical disease and associated mortalities reported in New South Wales, Victoria, Western Australia [6–8], and more recently in South Australia [9]. Other countries including New Zealand [10], Japan [11], China [12] and Korea [13] have also experienced outbreaks associated with *Theileria orientalis. T. orientalis* can be separated into several genotypes, namely, type 1 (Chitose), type 2 (Ikeda), type 3 (Buffeli), types 4–8 [4, 6] and types N1-N3 [14]. The emergence of the pathogenic genotype *T. orientalis* Ikeda, and its rapid spread in Australia is of increasing concern [2, 6].

An understanding of the modes of transmission is essential for a full appreciation of the disease epidemiology and for the rational formulation of control measures for the Australian outbreaks. It appears that *Haemaphysalis longicornis* is the likely biological vector tick in southern Australia [15], however unequivocal evidence for mechanical transmission of *T. orientalis* is lacking. A single attempt to transmit *Theileria* mechanically by *Stomoxys calcitrans* and by needle puncture failed in Australia [1]. However, although the precise details were not provided, *T. orientalis* is transmissible by blood inoculation (volumes and route unknown), inducing a febrile response with large numbers of parasites in the peripheral blood [1, 16]. It has been hypothesised that mechanical transfer of theilerial piroplasms by re-using vaccination needles or from the proboscis of biting flies could result in disease [17]. While confirmatory evidence is scarce, a Japanese study reported that a biting tabanid, *Tabanus trigeminus*, under “certain” (unspecified) conditions could mechanically transfer *Theileria* (cited in [18]), while a second study demonstrated mechanical transmission of *T. orientalis* using the sucking louse *Linognathus vituli* [18].

The development of reliable recommendations for livestock producers to counter disease outbreaks has been hampered by the lack of understanding of the parasite epidemiology and pathogenesis in Australia. Standard husbandry practices that include blood transfer can include re-using castration knives, vaccination/medication needles, ear notching and injury during transport and yarding. Current measures recommend washing and disinfecting castration knives, avoiding multiple use of needles or, where impractical for herd vaccination, to use sharp needles and change these regularly to minimise blood transfer [19].

This study is the first to examine whether blood inoculation can mechanically transfer an Australian strain of *T. orientalis* and the quantities that may be involved. This study is also the first to test the sucking louse *L. vituli* in Australia*,* and to examine samples of colostrum for the presence of *T. orientalis* Ikeda. The discussion addresses the epidemiological risks posed by animal husbandry practices and biting arthropods.

## Methods

### Animal ethics

This research was carried out in accordance and with the approval of the University of Sydney Animal Ethics Committee Project Number 673.

### Collection of inocula

A recumbent Angus steer exhibiting clinical signs of theileriosis (tachypnoea, tachycardia, pale mucous membranes, weakness), had a packed cell volume of 8 % and piroplasms on blood smear. Diagnosis of bovine theileriosis was confirmed by quantitative PCR (qPCR) and the genotype was confirmed as *T. orientalis* Ikeda [20].

Using a CPDA-1 single blood-pack unit (Fenwal International Inc.), 250 ml of blood was collected from the jugular vein of the affected steer. Once collected, 2.5 ml blood was decanted into each of 5 ml cryotubes and 2.5 ml of the cryopreservant polyvinylpyrrolidone (PVP 40,000; pH 7.2) was added. The mixture was then placed in the vapour phase of liquid nitrogen for 15 minutes before being lowered into the liquid nitrogen for storage.

A second, fresh inoculum was obtained from a 6 month old calf, diagnosed by qPCR as infected with *T. orientalis* Ikeda. This calf showed no obvious clinical signs of theileriosis apart from being much smaller than calves of the same age. One ml of blood was collected into each of three syringes containing CPDA-1 anticoagulant. This blood was not stored but was used immediately for inoculation into the three calves from Group 2.

The parasite levels in each inoculum were determined by qPCR as described below.

### Trial location and facilities

The research was conducted on a 3 acre property near Bairnsdale in Victoria (37°44’03.6”S 147°39’31.6”E) under typical field conditions for cattle in the Gippsland region. Animals were kept in under natural conditions to mimic the more strenuous physiological conditions experienced in the field versus pen studies. The property was away from any bushland and was bordered by house blocks on two sides, a road on one side and unstocked land on the other boundary. The trial was undertaken during Summer and Autumn and calves were monitored daily for the presence of ticks. Calves were acclimatised to the trial site for 2 weeks to become accustomed to the sheds, yards, crush and the hand feeding regime.

### Iatrogenic transmission study

For the iatrogenic transmission study, 13 bucket reared Holstein-Friesian steers aged 6 months were sourced from a commercial dairy herd near Bairnsdale in Victoria (37°49’27.1”S 147°31’47.3”E). All animals had been castrated, vaccinated, drenched and dehorned prior to arrival. Recipient calves (*n* = 9) and negative control calves (*n* = 3) tested negative for *T. orientalis* by qPCR within 10 days of birth and again upon re-testing immediately prior to the beginning of the trial. The positive control calf (a donor animal) tested positive for *T. orientalis* at the commencement of the study and was monitored by qPCR thereafter. All calves all tested negative for Bovine Viral Diarrhoea Virus by an ear notch test for antigen (IDEXX; BVDV Ag Point-of-Care Test). Calves were randomly assigned to one of four treatment groups. Group 1 calves (H1-H3) were inoculated intravenously with 20 mL of inoculum containing 10 mL cryopreserved, infected blood. Calves in Group 2 (M1- M3) each received 1 ml of fresh blood and those in Group 3 (Calves L1, L2, L3) each received 0.2 ml of inoculum, containing 0.1 ml cryopreserved blood. Group 4 contained three negative control calves and the positive donor calf. Negative control calves in this study acted as sentinel animals for active transmission by ticks and other possible vectors.

Cryopreserved blood was thawed rapidly in a water bath at 37 °C for 5 min before being transfused via the jugular vein into the recipients. Calves were yarded, clinically examined and bled for qPCR testing to ensure they were negative for *Theileria orientalis* prior to transfusion. Thirty minutes before inoculation of blood, each calf in all treatment groups received 100 mg of the antihistamine chlorpheniramine maleate (Histamil) by intramuscular injection, to lower the risk of any transfusion reaction. Animals were monitored for an hour following infusion for any adverse reactions during which time a post-inoculation blood sample was drawn.

To monitor the progress of the trial, calves were yarded daily. During these times, calves were hand fed (1.5 kgs Barastoc Heifer Developer Pellets) and observations of behaviour, gait, appetite and respiration were made. To detect theilerial infection, blood was collected from the tail veins of all calves into 5 ml EDTA blood tubes three times weekly for 13 weeks. Blood samples were stored at −20 °C until required for qPCR testing

### Investigation of lice as potential mechanical transmitters of *T. orientalis*

Following the conclusion of the iatrogenic transmission study, experimental calves were transported to a 119 acre property in Fernbank, Victoria (37°48'21.9"S 147°19'41.7"E). Approximately 2 months after transfer to this property (35 weeks after inoculation), 2 calves were found to be naturally infested with sucking lice. Lice from each animal were collected using flea combs and blood was collected from the same calves for qPCR testing.

### Colostral transmission study

Thirty cows from a commercial dairy herd within the *Theileria*-endemic region of Bairnsdale, Victoria were randomly selected. Colostrum was collected from each quarter into separate sterile containers within 24 hours of parturition. Samples were stored at −20 °C prior to qPCR and ELISA. EDTA blood was sampled from the 30 cows and their calves at 3–6 weeks of age. All samples were also tested via qPCR [20] and *T. orientalis* MPSP ELISA [21].

### ELISA antigen preparation

MPSP genes amplified from each of the *T. orientalis* Ikeda, Chitose and Buffeli MPSP types were cloned into the Champion pET100 D-TOPO expression vector (Invitrogen, Carlsbad, Calif., USA) according to the manufacturer’s instructions. Primers used for PCR amplification of the MPSP gene fragments were as follows (the CACC overhang required for directional cloning are underlined and the artificial stop codon for terminating translation is shown in bold): Ts-Bc: 5' CACC-TGC TCT GCA ACC GCA GAG 3', Ts-Cc: 5' CACC-TTC CTC ATC GTC TCT GCA ACT 3', Ts-Ic: 5' CACC-ATC GTC TCT GCT ACC GCC GC 3' and Ts-Rc: 5' CTA TGT GAG ACT CAA TGC GCC TA 3'. PCR products were amplified in 50 μL volumes using 1 × *Pwo* polymerase reaction buffer and 1U *Pwo* polymerase (Roche, Basel, Switzerland), 100 μM dNTPs and 10 μM primers. The Ts-Bc, Ts-Cc and Ts-Ic (forward) primers were each paired with the Ts-Rc (reverse) primer in amplification of the Ikeda, Chitose and Buffeli MPSP genes respectively. Antigen purification and dialysis was performed as previously described [22]. Antigen concentration was estimated using the bichinchoninic acid (BCA) assay (Thermo Scientific, Rockford, IL, USA). Stock antigens were stored at −20 °C as an equimolar cocktail of the 3 MPSP antigens.

### ELISA assay

ELISA plates (Linbro/Titertek E.I.A Cat no. 76-381-04, MP Biomedicals LLC., Santa Ana, CA, USA) were coated with 1 μg of antigen in 100 μL carbonate coating buffer (pH 9.6) per well and incubated overnight at room temperature (22 °C) in a humid chamber. All washing steps were performed as five washes with Tris-buffered saline (TBS) containing 0.05 % Tween 20 (TBST) using an automatic 96-well ELISA washer. After coating, plates were washed in TBST and then blocked with 1 % bovine serum albumin (BSA) in TBS for 1 h. After washing with TBST, 100 μL of each test serum sample and control sera diluted 1:100 in 1 % BSA-TBST were added to each well and incubated for 1 h. After washing in TBST, 100 μL of monoclonal anti-bovine IgG (clone BG-18 alkaline phosphatase conjugate, Sigma) diluted 1:20 000 in 1 % BSA-TBST was added to each well and incubated for 1 h. The plates were washed and developed with BluePhos alkaline phosphatase substrate (KPL). Plates were read 610 nm (A_610_) on an xMark plate reader (Bio-Rad Laboratories, Hercules, CA, USA). Optical density (OD) at 610 nm was determined for each plate when the positive control to negative control serum had a mean OD_610_ ratio of >5 and the OD_610_ of the negative serum was < 0.15. Results were expressed as an ELISA ratio (ER: mean OD test serum/mean OD of the negative control serum). Sera with an ER < 2 were considered negative and an ER ≥ 2 as positive. These results were shown previously to give a specificity of 98 % and sensitivity of 67 % in comparison to PCR testing [21].

### DNA extraction and qPCR

DNA was extracted from all blood and colostrum samples using the DNeasy Blood and Tissue kit (Qiagen, Alameda, California, USA) according to the manufacturer’s instructions and as described previously [20]. The same kit was used for DNA extraction from lice, but using the manufacturer’s tissue protocol. Prior to extraction, lice were pooled (~20 lice per pool) into 7 pools per animal and homogenized with sterile microfuge pestles. The remaining lice from each animal were homogenized and batch tested.

qPCR was performed using a validated multiplex qPCR assay for *T. orientalis* detection and genotype differentiation as described previously [20]. This assay was used for quantification of the infection intensity of *T. orientalis* (Universal) and to detect the Ikeda and Chitose genotypes (UIC assay). DNA extracts were also tested for the Buffeli genotype of *T. orientalis* using a quantitative singleplex qPCR (B assay) as described in [23].

## Results

### Iatrogenic transmission trial

**Fig. 1:**
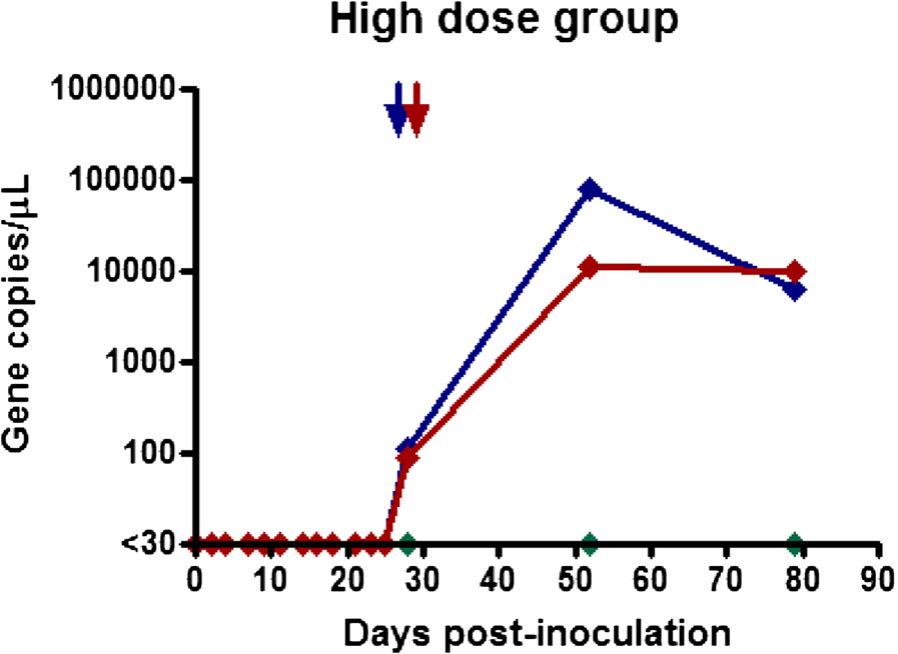
Gene copy number per microliter of blood over time post transfusion. Results for calves L1, L2, and L3 (Low-dose group) who received 0.1 mL of cryopreserved inoculum

qPCR was used to determine the total number of parasites inoculated into recipient calves. One MPSP gene copy was assumed to equate to one parasite given that the piroplasm stage of *T. orientalis* is haploid. The cryopreserved stabilate had a *T. orientalis* MPSP gene copy number of 1.25 × 10^5^, while that of the fresh inoculum was 1.5 × 10^5^ gene copies/uL. Both of the donor animals had infection intensities considered to be in the clinically relevant range [20]. The parasite dosages were as follows: Group 1 (H1-H3) = 1.25 x 10^9^ parasites; Group 2 (M1-M3) = 1.5 x 10^8^ parasites and Group 3 (L1-L3) = 1.25 x 10^7^ parasites. Eight of the nine inoculated calves became qPCR positive for theilerial DNA during the trial period. Calf H2, from the high dose cryopreserved blood group became clinically ill within 2 hours of transfusion. The calf became lethargic and recumbent for approximately 16 hours, exhibiting tachycardia, tachypnoea, and pyrexia (40.7 °C). This calf recovered but tested negative for theilerial DNA for the length of the trial. The remaining calves in the high dose group became positive for theilerial DNA at 28 days (4 weeks) following transfusion and remained positive until the end of the monitoring period (Fig. 1). The maximum infection intensity from this group was 7.8 × 10^5^ gene copies/μL of blood and corresponded to the Ikeda genotype.

**Fig. 2:**
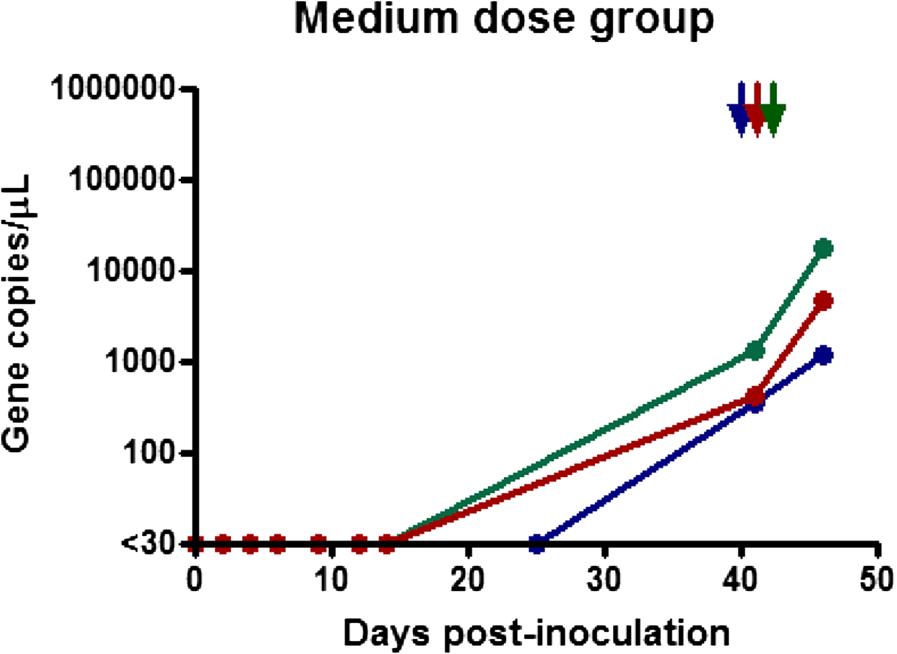
Gene copy number per microliter of blood over time post transfusion. Results for calves M1, M2, and M3 (Medium-dose group) who received 1 mL of fresh blood inoculum

**Fig. 3:**
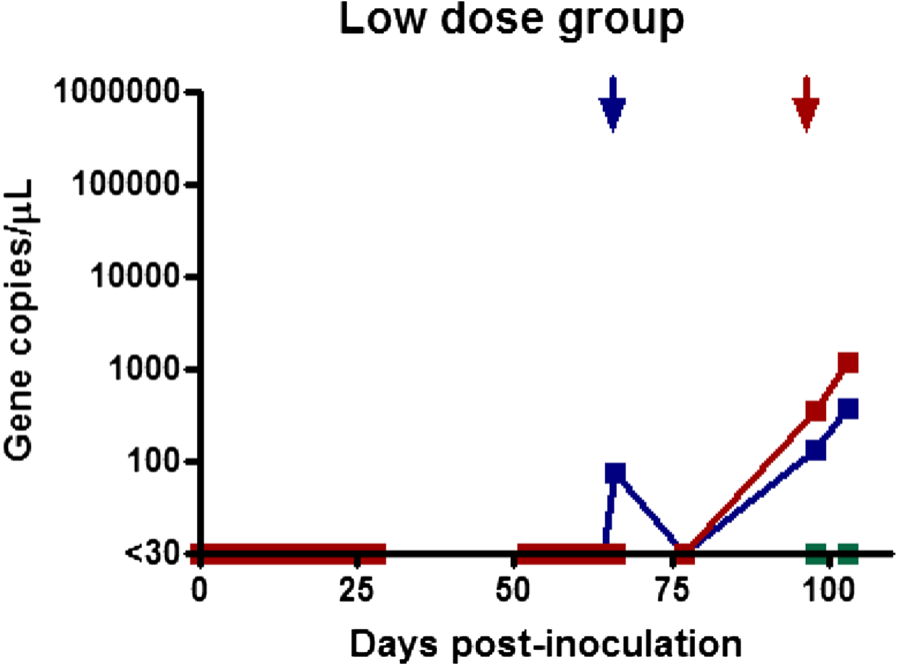
Gene copy number per microliter of blood over time post transfusion. Results for calves H1, H2, and H3 (High-dose group) who received 10 mL of cryopreserved inoculum

**Fig. 4:**
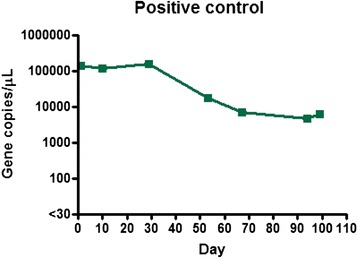
Gene copy number per microliter of blood over time. Results for the positive control calf throughout the trial period

Calves in the medium dose group (1 ml of fresh blood) all became positive at 41 days (almost 6 weeks) following transfusion and remained positive until the end of the monitoring period (Fig. 2). The maximum infection intensity for this group was 1.7 × 10^5^ gene copies/μL and corresponded to the Ikeda genotype. Calves in the low dose group (0.1 ml of cryopreserved blood) also became positive (Fig. 3). Calves L1 and L2 became positive at day 66 (9.5 weeks), while calf L3 was first positive at day 98 (14 weeks). The maximum infection intensity for this group was 1.2 × 10^3^ gene copies/μL of blood and corresponded to the Ikeda genotype. All negative control calves remained negative throughout the trial period and daily examination did not reveal ticks on any animals throughout the trial period. The positive control calf remained positive throughout trial, although the infection intensity changed throughout the trial period (Fig. 4).

### qPCR detection of *T. orientalis* in *Linognathus vituli*

Lice collected around 35 weeks after inoculation, from calves transferred to Fernbank, Victoria were identified morphologically as the sucking louse *L. vituli.* Blood collected from the 2 infested calves were positive for *T. orientalis* with infection intensities of 1.3 × 10^5^ and 2.2 × 10^5^ gene copies/μL of blood. Only the Ikeda genotype of *T. orientalis* was detected. Similarly, 14 of the 16 lice pools, including the batch tested lice were positive for *T. orientalis,* while the remaining two pools were just below the diagnostic threshold of 30 gene copies per μL of DNA extract. All lice pools were positive for the Ikeda genotype and negative for the Chitose and Buffeli genotypes. Parasite levels with the lice DNA extracts ranged from 37–362 gene copies/μL of DNA extract.

### *T. orientalis* and *T. orientalis* MPSP antibodies in colostrum

Using qPCR, 4/30 colostrum samples tested positive for *T. orientalis* (Table 1)*.* In addition to these, six samples of colostrum tested positive for theilerial antibodies using the *T. orientalis* ELISA. Six calves received antibodies in colostrum and from these, only one tested positive for MPSP antibodies. Calves that received qPCR-positive colostrum showed no evidence of *T. orientalis* infection. While no calves tested positive by qPCR for *T. orientalis,* 16 of the 30 dams were *T. orientalis* positive*.* Of these 16 cows, 67 % were positive for Ikeda genotype only, 25 % had a mixed infection of Ikeda and Buffeli, and one dam harboured Chitose only (Table 1).

**Table 1:** Colostral transmission trial results. qPCR and ELISA results on colostrum, and bloods from dams and calves

Cow ID	Colostrum Universal	Colostrum qPCR	Colostrum ELISA	Dam Universal	Dam ELISA	Calf qPCR	Calf ELISA
783	0	-	+3.26	14638 I, B	1.86	N/A	N/A
733	0	-	-	0	-	0	-
698	0	-	-	<T	-	0	-
633	24	-	1.88	<T	1.88	0	-
630	0	-	-	0	-	0	-
806	0	-	+2.10	1067 C	-	N/A	N/A
635	33	+ (I-39)	+2.07	322 I	-	N/A	N/A
661	5	-	-	0	-	0	-
851	0	-	-	916 I	-	0	-
742	41	+ (I-48)	+2.42	1691 I	-	0	1.65
605	8	-	-	244 I, B	-	0	-
670	0	-	-	<T	-	N/A	N/A
869	0	-	-	0	-	N/A	N/A
615	86	+ (I-83, B-99)	-	2143 I, B	-	0	-
487	0	-	-	0	-	N/A	N/A
708	0	-	-	0	-	N/A	N/A
710	572	+ (I-576, B-999)	+2.35	5407 I, B	-	N/A	N/A
753	11	-	-	10607 I	-	N/A	N/A
647	0	-	-	<T	-	N/A	N/A
518	0	-	-	0	-	N/A	N/A
776	17	-	+4.35	5440 I	+2.98	0	+3.90
767	3	-	-	1985 I	-	0	-
804	0	-	-	0	-	0	-
838	2	-	-	4593 I	-	0	-
602	3	-	-	2489 I	-	0	-
866	0	-	-	1863 I	-	0	-
650	0	-	-	0	-	0	-
832	3	-	-	44 I	1.81	0	-
747	5	-	-	453 I	-	N/A	N/A
732	3	-	-	<T	-	0	-

I = Ikeda, C = Chitose, B = Buffeli, T = Threshold + =Positive result, − = Negative result, N/A = Male calf not available to retest as they had been sold by the time of blood testing, Units = Gene copies/uL

## Discussion

The results of this study have shown that *T. orientalis* infections can be readily established after inoculation of fresh or cryopreserved, infected bovine blood and persist for at least 5 months. Fresh blood was used as a medium dose group in an attempt to determine if the cryopreservation process used for the high and low dose groups affected the infectivity of the inoculum. The relationship between inoculated blood volume and time to patency clearly indicated the inverse dose- response and that the cryopreservation process preserved the viability of the piroplasms. Vector transmission did not occur as the three control calves remained negative for *T. orientalis* throughout the trial period [20]. Only one inoculated calf did not become *Theileria* qPCR positive during the trial period (calf H2). This calf was thought to have suffered from a transfusion reaction and became lethargic and pyrexic soon after transfusion. Although this calf recovered within 24 hours, it remained negative for *T. orientalis* possibly resulting from parasite clearance during the pyrexia.

The peak infection intensity for each treatment group corresponded to a low to moderate *T. orientalis* infection. From the level of donor parasitism (around 2 %), the transfer of around 10^7^ parasitised erythrocytes (in 0.1 ml) can establish a parasitosis with sufficient quantum to be detected and graded by qPCR as a low-level infection. The genotype Ikeda was the sole genotype detected at each of these peak periods, corresponding to the genotype present in the inocula, further confirming the lack of tick transmission. A number of studies have suggested that the dominant parasite population can change [23, 24], during persistent infection in cattle over several months [25]. Vector and host immunological factors are thought to result in genotype switching and this is likely to play a role in parasite persistence within mammalian hosts and its transmission from tick vector [23, 25]. Although genotypic switching was not observed in this study, it could have a role in the persistence and spread of theileriosis.

Routine husbandry practices whereby blood is transferred between animals is a concern for the mechanical transmission of *Theileria* without the need for an intermediate host. We have shown that *T. orientalis* can be transmitted by low numbers of piroplasms, something that might also occur iatrogenically. This may include transfer of piroplasms when re-using needles between cattle (e.g. vaccinations), contaminated castration knives, ear notching procedures, as well as injury sustained during yarding and transport of cattle. The risk of iatrogenic transmission would depend on the volume and parasitaemia of the blood transferred and on the ability of the parasite to survive outside a host before being inoculated into a susceptible animal.

Mechanical transmission through biting arthropods is a possible mode of transmission of *T. orientalis,* as has been demonstrated for African trypanosomes [26]. Hematophagous insects, including flies, lice and mosquitoes as well as non-biological vector ticks could have the potential to transmit infection*.* Regurgitation of part of a previous blood meal or passive transfer of blood on mouth parts are possible modes of transmission. It has been shown that approximately 1.3 % of gut content in one species of tick can be regurgitated during a blood meal [27], leading to possible transfer for piroplasms. *Stomoxys* (Diptera: Muscidae) are obligate blood-sucking insects that are known mechanical vectors for viral, bacterial, rickettsial, protozoal, and helminth pathogens [28]. A recent study failed to detect *T. orientalis* in biting flies, however *T. orientalis* was detected in mosquitoes [15]. Blood meals from mosquitoes are in the order of 1 nl to 6 μl [29], so the risk of transmission would depend on both the level of parasitaemia in the donor and the cumulative volumes inoculated or transferred by the biting arthropods.

The sucking louse of cattle, *(L. vituli)* is a widespread parasite in southern Australia. Here, we present the first evidence that Australian populations of *L. vituli* can carry detectable amounts of *T. orientalis* Ikeda. *L. vituli* has been shown to transmit *Theileria* to splenectomised calves when previously fed on infected cattle that had been inoculated with a sporozoite suspension [18, 26]. The transmission was shown to be mechanical via regurgitation and not biological [18]. This conclusion was also supported indirectly by studies where mechanical transmission with male ticks was attempted but was not successful [30]. Fujisaki (1993) showed detectable theilerial piroplasms in calves at 49 days and 33 days after lice were placed on the calves in separate experiments after feeding on calves with a parasitaemia of 1.8−6.5 % and 3.1−4.4 % respectively [18]. This was a similar prepatent period to the high dose inoculum in the current study. While splenectomy undoubtedly facilitated the establishment of the louse-borne infection as did intravenous inoculation in our trial, both confirmed the ability to transfer the parasite with piroplasmic stages of *T. orientalis.*

Although we have shown the minimum infectious dose of *T. orientalis* is very low, the risk of mechanical spread from biting arthropods would be highly dependent on several principal factors to provide a sufficient quantum of piroplasms to initiate a detectable (and transferrable) infection. These would include the prevalence of infection in the herd and the parasitaemia of infected donors, the volume of blood inoculated into the naïve host, the parasite load from the previous blood meal and the number of biting arthropods. Finally, the ability of *T. orientalis* to remain viable between feeds and the subcutaneous inoculation of this blood would also affect the chances of transmission, as would the ability of flying arthropods (flies and mosquitos) to find a recipient in a short period of time or to be transferred by contact (lice).

Mechanically-transferred parasitoses have been shown to produce viable infections in ticks which subsequently developed sporozoites in their salivary glands after feeding on an inoculated calf [18]. The sexual phase of the *T. orientalis* lifecycle occurs within vector ticks, allowing genetic recombination to occur. The genetic diversity generated within the parasite population by passage through insect vectors is believed to be an important immune evasion mechanism in a number of apicomplexans including the theileriae [31–33]. However, mechanical transmission results in the direct transfer of haploid phase piroplasms from host to host, thereby bypassing the sexual phase of the lifecycle. The inability of the parasite to genetically recombine therefore would be expected to reduce overall diversity within parasite population. Thus, extensive mechanical transfer of the parasite would be expected to decrease the ability of the parasite to evade the host immune system. Therefore, since the parasitosis persists in the recipient, mechanical transmission may help to explain the rapid spread of the parasite over large geographical areas. However, it is likely that biological vector ticks are essential to maintain virulence and pathogenicity.

Given the small volume of blood required for the transmission of *T. orientalis,* other biological modes of transmission need to be considered. One such method is the transmission between dams and their calves. Aside from transplacental transmission which has been reported to occur, at least experimentally [13], transmission could conceivably occur through blood transfer in colostrum. This study is the first to report the presence of *T. orientalis* in colostrum. We have shown that colostrum can contain clinically-significant quantities of parasites as determined by qPCR analysis. Of the 30 samples of colostrum tested, only 4 contained significant levels of *T. orientalis,* and calves receiving this colostrum within 6 hours of birth failed to become positive. From the samples collected on this commercial dairy farm, 53 % of cows tested positive for *T. orientalis.* Erythrocytes are commonly present in colostrum within the first 24 hours from birth, however the fact that only a small number of positive dams contained *T. orientalis* in their colostrum is likely due to the fact that the negative colostrum samples simply did not contain blood.

The separation of the maternal and foetal blood supplies by the epitheliochorial placentation in cattle prevents transfer of macromolecules into the foetal circulation. However, immunoglobulins, cytokines, leukocytes, and functionally viable maternal cells (possibly including memory T cells), have all been demonstrated to be transferred via colostrum into the neonatal bloodstream [34, 35]. Parasitised red cells could conceivably transfer into a calf within 24 hours of birth, releasing piroplasms. The capacity for ingested piroplasms to transfer into recipient erythrocytes is not known, but macroschizonts of *Theileria parva* within cultured lymphoblasts can transfer into recipient lymphocytes with an efficiency of around 10 ^−5^ when inoculated into fully allogenic (unrelated) recipients [36]. Therefore it may be possible for other life stages of *T. orientalis* to be transmitted via colostrum. Although no calves tested positive for *T. orientalis* when tested at 3–6 weeks of age, the number of positive colostrum samples transferred to calves in this trial was insufficient to exclude the possibility of colostral transmission of piroplasms.

This is the first study to examine the presence of antibodies in colostrum and the possible transfer to calves. Six of the 30 samples contained *T. orientalis* antibodies, whereas blood from only 2 cows was positive by ELISA. Colostrum is known to contain higher concentrations of immunoglobulins than serum [37–39]. One of the 30 cows tested positive by qPCR for *T. orientalis* in both blood and colostrum. This cow also had antibodies in both serum and colostrum, and her 4-week old calf was the only recipient testing positive for *T. orientalis* antibodies. Although the numbers investigated in this trial was small, detectable levels of theilerial antibodies can be transmitted via colostrum to calves if the original titre is sufficiently high and calves are bled before 3 weeks old, after which time maternal antibody titres decline rapidly. Whether these maternal antibodies have an immunoprotective effect remains unclear.

## Conclusions

*T. orientalis* can be transmitted by low volumes of blood from infected to naïve animals. Although this mode of transmission did not result in clinical disease, parasitaemia can persist and allow for asymptomatic carriers to occur within herds for at least 5 months for possible ingestion by intermediate hosts or vectors. This raises the possibility of iatrogenic transmission through husbandry practices and the likelihood that biting arthropods can act as mechanical vectors as distinct from obligatory intermediate hosts. Mechanical transmission, including colostral transfer, might help to explain the rapid spread of *T. orientalis* and associated clinical outbreaks in southern Australia and elsewhere.

## Abbreviations

PCR: Polymerase Chain Reaction
qPCR: Quantitative Polymerase Chain Reaction
UIC: Universal Ikeda Chitose
